# Comparison of long-term bowel symptoms after laparoscopic radical hysterectomy versus abdominal radical hysterectomy in patients with cervical cancer

**DOI:** 10.1007/s00192-022-05351-x

**Published:** 2022-09-12

**Authors:** Ruiju He, Yiwei Xue, Xinrong Zhuang, Huizhong Wang, Ye Lu

**Affiliations:** 1 Department of Obstetrics and Gynecology, Peking University First Hospital, No. 1 Xi’anmen Street, Beijing, 100034 China; 2 The Affiliated Hospital of Chengde Medical College, Chengde, HeBei China

**Keywords:** Radical hysterectomy, Cervical cancer, Bowel symptoms

## Abstract

**Introduction and hypothesis:**

The objective of this study was to compare the long-term bowel symptoms between laparoscopic radical hysterectomy (LRH) and abdominal radical hysterectomy (ARH) in patients with cervical cancer.

**Methods:**

A total of 207 patients who underwent radical hysterectomy (79 underwent LRH and 128 underwent ARH) at Peking University First Hospital from January 2010 to August 2020 were enrolled and their bowel symptoms were investigated using the Colorectal Anal Distress Inventory-8 (CRADI-8) of the Pelvic Floor Distress Inventory-20. The prevalence and severity of bowel symptoms were compared in the LRH and ARH groups, and multivariate analysis was performed to determine the factors associated with bowel symptoms.

**Results:**

There was no difference in the CRADI-8 scores between the two groups. However, the prevalence of straining at stool was significantly higher in the ARH group than in the LRH group (19.5% versus 1.3%, *p*<0.001), and the score was significantly higher in the ARH group than in the LRH group too (0.4 versus 0, *p*<0.001). The prevalence of incomplete defecation was significantly higher in the ARH group than in the LRH group (13.3% versus 3.8%, *p*=0.029), and the ARH group also had a significantly higher score than the LRH group (0.3 versus 0.1, *p*=0.028). Multivariate analysis showed that ARH and postoperative interval were independent risk factors for the development of straining at stool.

**Conclusions:**

Patients with cervical cancer who underwent ARH may be more likely to develop symptoms related to constipation than those who underwent LRH. This finding has to be interpreted with caution owing to the study design.

## Introduction

The World Health Organization (WHO) lists cervical cancer (CC) as the fourth most common cancer in women, with an estimated 604,000 new cases and 342,000 deaths worldwide, and 110,000 new cases and 59,000 deaths in China alone in 2020 [1]. Radical hysterectomy combined with pelvic lymph node dissection is an effective treatment for early-stage CC [2]. Radical hysterectomy is performed using laparoscopic radical hysterectomy (LRH) or abdominal radical hysterectomy (ARH) [3]. Radical hysterectomy may damage the pubic nerves, pelvic nerves, and the lower abdominal plexus, and lead to pelvic floor dysfunction (PFD) [4]. Studies have shown that compared with ARH, LRH has the advantages of less intraoperative blood loss and shorter postoperative recovery time [5, 6], but there are a few studies that compared PFD after the two radical procedures. Most are on urinary symptoms and fewer are on bowel symptoms. Hwang et al. concluded that the incidence of the vesicovaginal fistula was higher in the laparoscopic group than in the open group [7]; a retrospective study in China comparing postoperative complications in 18,447 patients undergoing ARH or LRH for CC also showed that more patients developed vesicovaginal fistula in the LRH group [8]. A study by Laterza et al. [9] showed that the Wexner score for constipation 6 months after ARH was significantly higher than that before surgery, whereas the Wexner score for constipation after LRH was not different from that in the preoperative period. Based on the lack of data on bowel symptoms after LRH and ARH, we designed this study to explore the prevalence and risk factors for bowel symptoms after radical hysterectomy with two preliminary surgical approaches.

## Materials and methods

### Participants

This was a cross-sectional questionnaire study of patients with CC who underwent radical hysterectomy at the Peking University First Hospital between January 2010 and August 2020. We selected patients whose International Federation of Gynecology and Obstetrics (FIGO) staging of cancer of the cervix uteri was IA1 to IIA2. Patients with the following medical histories were excluded: previous mental diseases such as cerebrovascular disease sequelae, anxiety, depression, cognitive impairment; previous organic intestinal diseases such as inflammatory bowel disease and intestinal tumor; other malignant tumors; previous anorectal surgery; and recurrence and metastases of CC undergoing enterectomies and enterostomies.

This study was approved by the Ethics Committee of Peking University First Hospital, and informed consent was obtained via telephone before the survey.

### Methods

We used the Colorectal Anal Distress Inventory-8 (CRADI-8) of the Pelvic Floor Distress Inventory-20 (PFDI-20) [10] to assess postoperative bowel symptoms in patients with CC. This questionnaire is a level A questionnaire recommended by The International Consultation on Urological Diseases [11] and is used widely in clinical practice. The reliability and validity of the questionnaire in the simplified Chinese version have been verified in China [12]. The PFDI-20 consists of three subscales with 20 questions. The three subscales are the Pelvic Organ Prolapse Distress Inventory 6, the CRADI-8 and the Urinary Distress Inventory 6. Each question is scored on a scale of 0–4 and the individual subscale scores (0–100) are the mean scores of the questions multiplied by 25. Higher scores indicate a greater impact on quality of life, and our study used the CRADI-8. Four trained pelvic floor specialists investigated the patients’ bowel symptoms in the last 3 months. Demographic and perioperative data were obtained from the medical record system of the Peking University First Hospital.

### Statistical analysis

The software IBM SPSS 24.0 was used for the statistical analysis. Descriptive statistics were used. The data were tested for normality according to the Shapiro–Wilk test. The Mann–Whitney *U* test was used to compare bowel symptom scores and the Chi-squared test was used to compare the prevalence of bowel symptoms. The independent variables were analyzed first using univariate analysis, followed by a binary logistic regression analysis to explore the risk factors for the postoperative bowel symptoms. Any variable for which the univariate test had a *p* < 0.15 was considered as a candidate for the binary logistic regression analysis. All the tests were two-sided, and the differences were considered to be statistically significant at *p* < 0.05.

## Results

From January 2010 to August 2020, a total of 935 patients with CC underwent radical hysterectomy at Peking University First Hospital. Among them, 483 patients were excluded from the study owing to the tumor stage being > IIA2, extra-fascial hysterectomy or modified radical hysterectomy, tumor recurrence, concomitant malignancy at other sites, intestinal obstruction, previous anorectal surgery, and enterostomy. The questionnaire was conducted on 452 patients and 301 (66.6%) responded, of whom 86 declined to participate owing to privacy considerations and being busy. Furthermore, 8 patients died, and 207 were eventually enrolled in the study. Among these patients, 79 (38.2%) underwent LRH, 128 (61.8%) underwent ARH, and no LRH procedure was converted to open surgery. Age, BMI, and parity of the two groups were comparable (Table 1). The mean postoperative interval was 3 (range 1–8) years in the LRH group and 5 (range 1–11 years) years in the ARH group.

**Table 1:** Demographic characteristics

Characteristics	LRH group (*N*=79)	ARH group (*N*=128)	*p* value
Age at surgery (years)	47.8±9.3	46.8±8.3	0.421*
Body mass index (kg/m^2^)	24.4 (17.7, 32.7)	24.2 (15.8, 36.0)	0.484**
Parity (times)	1 (0, 5)	1 (0, 4)	0.376**
Delivery mode, *n* (%)			0.672***
Vaginal delivery	62 (78.5)	98 (76.6)	
Cesarean section	9 (11.4)	19 (14.8)	
Both the above	3 (3.8)	2 (1.6)	
No history of childbirth	5 (6.3)	9 (7.0)	
Occupation, *n* (%)			0.102***
Office clerk	16 (20.3)	30 (23.4)	
A housewife	41 (51.9)	43 (33.6)	
Self-employed	10 (12.7)	17 (13.3)	
Farmer	0	4 (3.1)	
Retired	6 (7.6)	17 (13.3)	
Unclear	6 (7.6)	17 (13.3)	
Previous medical history, *n* (%)			0.221***
Hypertension only	11 (13.9)	15 (11.7)	
Diabetes only	1 (1.3)	0	
Both	4 (5.1)	2 (1.6)	
None	63 (79.7)	111 (86.7)	
History of abdominal surgery, *n* (%)	29 (36.7)	33 (25.8)	0.058***
History of cesarean section	14 (17.7)	20 (15.6)	

Data are expressed as mean±standard deviation, mean (range), or number (%)

*LRH* laparoscopic radical hysterectomy, *ARH* abdominal radical hysterectomy

*Independent samples *t* test, **Mann–Whitney *U* test, ***Fisher’s exact test

The FIGO stage and pathological type of the two groups were comparable. The duration of surgery was longer in the ARH group than in the LRH group, and there was more intraoperative blood loss in the ARH group than in the LRH group. As for adjuvant therapy, 26 (32.9%) had radiotherapy or chemoradiotherapy in the LRH group and 44 (34.4%) in the ARH group accordingly, with statistically significant differences (Table 2).

**Table 2:** Characteristics of oncology and surgery

Characteristics	LRH group (*N*=79)	ARH group (*N*=128)	*p* value
FIGO stage, *n* (%)			0.484*
IA2	4 (5.1)	4 (3.1)	
IB+IIA	75 (94.9)	124 (96.9)	
Pathological type, *n* (%)			0.117**
Squamous cell	66 (83.5)	117 (91.4)	
Nonsquamous cell	13 (16.5)	11 (8.6)	
Duration of surgery (min)	226 (144, 432)	168 (100, 358)	<0.001***
Blood loss (ml)	99 (20, 500)	229 (50, 1000)	<0.001***
Lymphadenectomy, *n* (%)	78 (98.7)	126 (98.7)	1.000*
Postoperative lymphocyst, *n* (%)	6 (7.6)	19 (14.8)	0.545*
Ovarian preservation, *n* (%)	29 (36.7)	49 (38.3)	0.883**
Adjuvant therapy, *n* (%)			0.012**
None	34 (43.0)	32 (25.0)	
Chemotherapy only	19 (24.1)	52 (40.6)	
RT or CRT	26 (32.9)	44 (34.4)	
Days in hospital	14 (6, 24)	15 (7, 53)	0.153***
Postoperative interval (years)	3 (1, 8)	5 (1, 11)	0.005***

Data are expressed as mean (range), or number (%)

*LRH* laparoscopic radical hysterectomy, *ARH* abdominal radical hysterectomy, *FIGO* International Federation of Gynecology and Obstetrics; *RT* radiation therapy, *CRT* chemoradiotherapy

*Fisher’s exact test, **Chi-squared test, ***Mann–Whitney *U* test

Bold text indicates statistical significance

There were 25 patients (19.5%) in the ARH group with straining at stool, significantly more than 1 patient (1.3%) in the LRH group (*p* < 0.001), and 17 patients (13.3%) in the ARH group with incomplete defecation, significantly more than 3 patients (3.8%) in the LRH group (*p* = 0.029). However, there was no difference in the overall prevalence of intestinal symptoms (*p* = 0.213; Table 3).

**Table 3:** Comparison of the prevalence of bowel symptoms after laparoscopic or abdominal radical hysterectomy

Bowel symptoms, *n* (%)	LRH group (*N*=79)	ARH group (*N*=128)	*p* value
Patients with one or more bowel symptoms	12 (15.2)	29 (22.7)	0.213*
Patients with subitem bowel symptom			
Straining at stool	1 (1.3)	25 (19.5)	<0.001*
Incomplete defecation	3 (3.8)	17 (13.3)	0.029*
Could not control defecation when the stool was formed	0	1 (0.8)	1.000**
Could not control defecation when the stool was loose	1 (1.3)	0	0.382**
Usually could not control flatus	1 (1.3)	0	0.382**
Defecation pain	1 (1.3)	7 (5.5)	0.158**
Urgent defecation	5 (6.3)	3 (2.3)	0.264**
Bowel bulge outside after defecation	2 (2.5)	11 (8.6)	0.137**

Data are expressed as mean (range)

*LRH* laparoscopic radical hysterectomy, *ARH* abdominal radical hysterectomy

*Chi-squared test, **Fisher’s exact test

Bold text indicates statistical significance

The ARH group had significantly higher scores in straining at stool than the LRH group (0.4 versus 0, *p* < 0.001); the ARH group had significantly higher scores in incomplete defecation than the LRH group (0.3 versus 0.1, *p* = 0.028); however, there was no difference in CRADI-8 between the two groups (Table 4).

**Table 4:** Comparison of bowel symptoms score after laparoscopic or abdominal radical hysterectomy

Bowel symptoms score	LRH group (*N*=79)	ARH group (*N*=128)	*p* value*
Total bowel symptom score (CRADI-8)	1.0 (0, 18.8)	3.4 (0, 34.4)	0.092
Subitem bowel symptom score			
Straining at stool	0 (0, 1.0)	0.4 (0, 3.0)	**<0.001**
Incomplete defecation	0.1 (0, 4.0)	0.3 (0, 3.0)	**0.028**
Could not control defecation when the stool was formed	0 (0, 0)	0 (0, 2.0)	0.432
Could not control defecation when the stool was loose	0 (0, 2.0)	0 (0, 0)	0.203
Usually could not control flatus	0 (0, 1.0)	0 (0, 0)	0.203
Defecation pain	0 (0, 2.0)	0.1 (0, 3.0)	0.128
Urgent defecation	0.1 (0, 2.0)	0.1 (0, 3.0)	0.157
Bowel bulge outside after defecation	0 (0, 1.0)	0.1 (0, 3.0)	0.072

Data are expressed as mean (range)

*LRH* laparoscopic radical hysterectomy, *ARH* abdominal radical hysterectomy

*Mann–Whitney *U* test

Bold text indicates statistical significance

Any variable for which the univariate test had a *p* < 0.15 was considered a candidate for binary logistic regression CCsis. Variables in the regression CCsis include surgical approach, postoperative interval ovarian preservation, intraoperative blood loss, duration of surgery, and days in the hospital. It suggested that ARH might be an independent risk factor for straining at stool (OR = 12.429, 95% CI: 1.311–117.828), and the risk for straining at stool after ARH was 12.4 times higher than after LRH. For each 1-unit increase in postoperative interval, the risk for straining at stool increased by 31.0% (Table 5).

**Table 5:** Analysis of factors affecting symptoms of straining at stool after radical hysterectomy

Variables		Straining at stool (*N*=25)	
		OR (95% CI)	*p* value
Surgical approach	LRH	1	
	ARH	12.429 (1.311–117.828)	**0.028**
Ovarian preservation	No	1	
	Yes	2.004 (0.779–5.152)	0.149
Blood loss (ml)		1.001 (0.998–1.003)	0.566
Duration of surgery (min)		1.002 (0.990–1.013)	0.770
Days in hospital		1.019 (0.948–1.096)	0.606
Postoperative interval (years)		1.310 (1.108–1.548)	**0.002**

*LRH* laparoscopic radical hysterectomy, *ARH* abdominal radical hysterectomy *OR* odds ratio, *95% CI* 95% confidence interval

Bold text indicates statistical significance

## Discussion

Patients with early-stage CC can survive for many years after surgery and adjuvant therapy, but their pelvic floor function may be impaired after treatment [13, 14]. Bowel and bladder dysfunction after a radical hysterectomy for CC may be associated with intraoperative injuries, which may destroy most pelvic autonomic nerves[15] and disrupt the reflex arc of the spinal cord controlling rectal emptying, leading to clinical symptoms [16]. However, it is uncertain whether different surgical approaches lead to differences in postoperative pelvic floor function.

In our study, there was no difference in long-term bowel symptom scores between the ARH group and the LRH group, but patients who underwent ARH were more likely to have difficulty defecating than those who underwent LRH. The binary logistic regression CCsis showed that the risk for straining at stool was 12.4 times higher for abdominal surgery than for laparoscopic surgery, and the prevalence of incomplete defecation was also higher in the ARH group than in the LRH group. Constipation includes straining at stool and incomplete defecation [17]. From this perspective, the results of this study are similar to those of existing studies [18]. They found that constipation scores were significantly higher at 6 months after surgery in the ARH group than in the preoperative period, but there was no change in the LRH group compared with the preoperative period, which may have been the case, because, compared with a radical abdominal hysterectomy, during laparoscopic surgery the posterior uterosacral ligament was retained and reduced damage to the innervated rectal nerves [19]. Furthermore, laparoscopic surgery has a greater magnification and field of view, resulting in less tissue destruction and less parametrial tissue containing nerves being removed.

Both the efficacy and complications of radical hysterectomy of CC are of concern. LRH is thought to have the advantages of less intraoperative bleeding and faster postoperative recovery than ARH [20], and the risk for straining at stool and incomplete defecation was lower in this study than in the ARH group. However, some research results have reported differences in oncological outcomes, and some studies have suggested that minimally invasive procedures, including LRH, might have lower disease-free survival than ARH [21, 22]. Furthermore, existing studies have concluded that the risk for ureteral injury and postoperative vesicovaginal fistula is higher in the LRH group than in the ARH group. Therefore, LRH should be chosen with caution in this regard. In this study, binary logistics regression showed that a longer postoperative interval is the risk factor for having intestinal symptoms related to constipation in CC patients, suggesting that in addition to the surgical approach, the postoperative interval might also be a factor affecting intestinal symptoms. However, owing to the limitations of the study design, it is not possible to draw a definite conclusion for the time being. To further confirm these conclusions, we need to design prospective cohort studies with baseline assessment and close follow-up in the future.

Several studies compared the efficacy and short-term postoperative complications of ARH and LRH, but there were very few studies on long-term postoperative bowel symptoms. In this study, a large sample size was used to research long-term postoperative bowel symptoms in patients with CC, and the intestinal symptoms of patients with radical hysterectomy by different surgical approaches were compared and analyzed, suggesting that there might be differences in long-term postoperative bowel symptoms between them. However, there are some limitations to this study too. This was a cross-sectional retrospective questionnaire study and some patients experienced a long postoperative interval, which may lead to recall bias; as all surveys were conducted by phone and questionnaires were completed by the researcher, there may be follower bias too.

In summary, there was no difference in the total postoperative long-term bowel symptom scores between the ARH group and the LRH group, whereas patients who underwent ARH were more likely to develop symptoms related to constipation than those who underwent LRH. Owing to the characteristics of the study design, this finding has to be interpreted with caution and prospective cohort studies need to be designed to continue to explore this topic in the future.
